# Transcriptional response to alcohol exposure in *Drosophila melanogaster*

**DOI:** 10.1186/gb-2006-7-10-r95

**Published:** 2006-10-20

**Authors:** Tatiana V Morozova, Robert RH Anholt, Trudy FC Mackay

**Affiliations:** 1 WM Keck Center for Behavioral Biology, North Carolina State University, Raleigh, NC 27695, USA; 2 Department of Zoology, North Carolina State University, Raleigh, NC 27695, USA; 3 Department of Genetics, North Carolina State University, Raleigh, NC 27695, USA

## Abstract

**Background:**

Alcoholism presents widespread social and human health problems. Alcohol sensitivity, the development of tolerance to alcohol and susceptibility to addiction vary in the population. Genetic factors that predispose to alcoholism remain largely unknown due to extensive genetic and environmental variation in human populations. *Drosophila*, however, allows studies on genetically identical individuals in controlled environments. Although addiction to alcohol has not been demonstrated in *Drosophila*, flies show responses to alcohol exposure that resemble human intoxication, including hyperactivity, loss of postural control, sedation, and exposure-dependent development of tolerance.

**Results:**

We assessed whole-genome transcriptional responses following alcohol exposure and demonstrate immediate down-regulation of genes affecting olfaction, rapid upregulation of biotransformation enzymes and, concomitant with development of tolerance, altered transcription of transcriptional regulators, proteases and metabolic enzymes, including biotransformation enzymes and enzymes associated with fatty acid biosynthesis. Functional tests of *P*-element disrupted alleles corresponding to genes with altered transcription implicated 75% of these in the response to alcohol, two-thirds of which have human orthologues.

**Conclusion:**

Expression microarray analysis is an efficient method for identifying candidate genes affecting complex behavioral and physiological traits, including alcohol abuse. *Drosophila *provides a valuable genetic model for comparative genomic analysis, which can inform subsequent studies in human populations. Transcriptional analyses following alcohol exposure in *Drosophila *implicate biotransformation pathways, transcriptional regulators, proteolysis and enzymes that act as metabolic switches in the regulation of fatty acid metabolism as important targets for future studies of the physiological consequences of human alcohol abuse.

## Abstract 2

Whole-genome transcriptional analysis following alcohol exposure in flies identifies key enzymes in conserved metabolic pathways that may contribute to human alcohol sensitivity.

## Background

The National Institute on Alcohol Abuse and Alcoholism has estimated that approximately 14 million people in the United States suffer from alcoholism [1]. Genetic studies have demonstrated that the propensity for alcohol abuse is determined by multiple genes. Thus, vulnerability to alcohol abuse shows all the hallmarks of a quantitative trait, as it is polygenic and subject to genotype by environment interactions. Efforts to map chromosomal regions (quantitative trait loci (QTL)) that harbor genes responsible for alcohol-related traits have identified at least 24 QTL regions in the mouse genome [2]. Although candidate genes have been identified within such QTL regions, including the multiple PDZ domain protein Mpdz on chromosome 4 [3], conclusive evidence that links candidate genes within QTL regions directly to the phenotype is often difficult to obtain. Quantitatively measuring alcohol dependence without confounding contributions of other psychiatric or social disorders is challenging with human subjects, although studies in ethnically defined populations have implicated alcohol dehydrogenase [4], the GABA_A _receptor complex [5], and the serotonin 1B receptor in alcohol sensitivity [6,7].

Flies are naturally exposed to ethanol, as they feed on fermented food. Exposing flies to low concentrations of ethanol stimulates locomotor activity, whereas high concentrations of ethanol induce an intoxicated phenotype that shows marked similarities to human alcohol intoxication, characterized by locomotor impairments, loss of postural control, sedation [8,9] and exposure-dependent development of tolerance [10]. Alcohol sensitivity in *Drosophila melanogaster *can be quantified in an 'inebriometer', a 122 cm long vertical glass column, which contains a series of slanted mesh partitions to which flies can attach [11]. Flies are introduced in the top of the column and exposed to ethanol vapors. As they lose postural control they fall through the column. The elution time from the column is used as a measure of sensitivity to alcohol intoxication. Following an initial exposure to alcohol, flies develop tolerance, manifested upon a second exposure by a shift in the elution profile [10]. Tolerance peaks within hours after exposure and persists up to 24 hours in some individuals [10].

Here, we exploited the power of the *Drosophila *system to evaluate whole-genome transcriptional profiles that reflect alterations in gene expression upon exposure to alcohol and during development of tolerance, to gain insights into the underlying cellular and physiological mechanisms that respond to alcohol exposure. We then evaluated alcohol sensitivity and the development of tolerance in flies with mutations in 20 genes that were implicated by the analysis of differential transcript abundance, and identified 15 novel candidate genes affecting alcohol related phenotypes, 10 of which have human orthologues. Thus, whole genome transcriptional analysis following alcohol exposure in *Drosophila *can identify candidate genes and metabolic pathways that are relevant to alcohol abuse and its physiological consequences in people.

## Results and discussion

**Figure 1:**
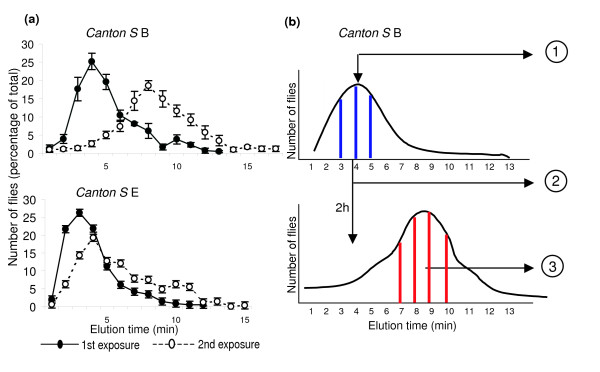
Sensitivity and development of tolerance to alcohol in *Drosophila melanogaster*. **(a) **Inebriometer elution profiles of *Canton S *B and *Canton S *E flies after exposure to concentrated ethanol vapor. Note the shifts in peak elution time between the first (solid symbols) and second (open symbols) ethanol exposure, and the difference in this peak shift between *Canton S *B and *Canton S *E. **(b) **Schematic diagram of the experimental design used to assess whole genome transcriptional changes after ethanol exposure. The numbers indicate samples collected for microarray analysis, as follows: 1, control flies; 2, flies after a single exposure to ethanol; 3, flies that develop tolerance during a 2 h interval prior to a second exposure to ethanol.

**Figure 2:**
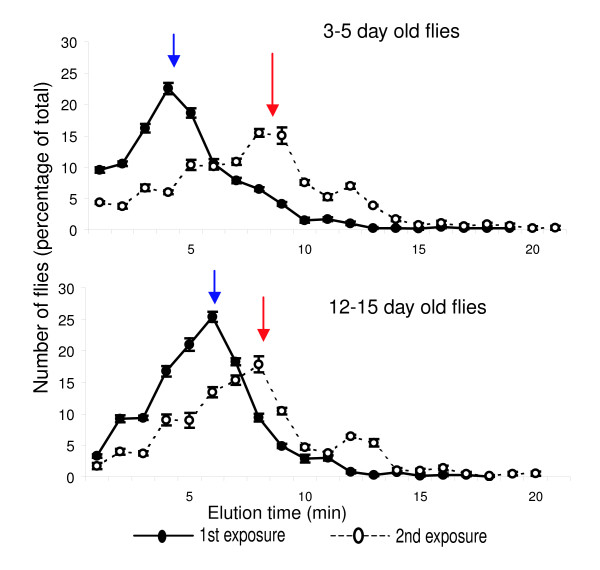
Inebriometer elution profiles of 3- to 5-day old (top) and 12- to 15-day old (bottom) flies after the initial (closed symbols, blue arrows) and a second, 2 h later (open symbols; red arrows) exposure to ethanol.

We hypothesized that analysis of gene expression after one and two exposures to ethanol would reveal novel genes and pathways underpinning the genomic response to ethanol, and that subsequent functional tests with mutations in such genes would confirm them as candidate genes for future detailed investigation. This strategy requires that mutations are generated in a common homozygous (isogenic) genetic background, since effects of segregating variants in an outbred strain will be of the same magnitude as the mutational effects we wish to detect. Fortuitously, a panel of single *P*-transposable element insertional mutations has been generated in each of five *Canton-S *derived isogenic strains (*Canton S *A, B, C, E, F) as part of the *Drosophila *Gene Disruption Project [12]. We took advantage of this resource and characterized for each of the 5 isogenic strains the responseto ethanol after an initial exposure and the development of tolerance after a second exposure 2 hours later, at 2 ages (3 to 5 days and 12 to 15 days post eclosion). We observed that sensitivity and development of tolerance are highly sensitive to genetic background (Additional data file 5). *Canton-S *B flies show the most pronounced development of tolerance, which is maximal two hours after exposure to undiluted ethanol vapors. Tolerance is least apparent in flies in the *Canton-S *E genetic background (Figure 1a). Furthermore, three- to five-day old flies are more sensitive than older flies to the initial alcohol exposure, and consequently show greater tolerance (Figure 2). Therefore, we used three- to five-day old *Canton-S *B flies for our transcriptional analysis.

We collected flies either without exposure to ethanol, immediately after exposure to ethanol and passage through the inebriometer, or from a population that had developed tolerance two hours after the initial ethanol exposure (Figure 1b). We used 5 independent replicates of 30 3- to 5-day old males for each time point and generated cRNA for hybridization to high density oligonucleotide microarrays. Raw microarray data are given in Additional data file 1.

We performed analysis of variance to identify probe sets with significant differences in expression between the three treatments, and a false positive discovery rate of *q *< 0.05 to account for multiple tests [13]. Significant probe sets were further analyzed by *post hoc *Tukey tests to identify which samples accounted for transcriptional differences between the treatments (Additional data file 2). We identified 582 probe sets with changes in gene expression between the treatment groups at *q *< 0.05 (Additional data file 2). Among these genes 112 encode predicted transcripts of unknown function, 173 have both mouse and human orthologues, 13 genes have orthologues only in the mouse, and 21 have only human orthologues. A total of 34 probe sets were significant at a false positive discovery rate of *q *< 0.001; of these, 8 have mammalian orthologues (Table 1).

We categorized the 582 significant probe sets as 'acutely' up- or down-regulated if transcript levels show sustained altered expression after the first exposure to ethanol; 'slowly' up-or down-regulated if transcript abundance only changes after the second exposure to alcohol; and 'transiently' up- or down-regulated if transcript levels are altered following the first exposure to ethanol, but are not sustained during the development of tolerance. We found 78 genes that show acute down-regulation and 77 genes that show acute up-regulation, In addition, 104 genes are slowly down-regulated, 258 genes are slowly up-regulated, 33 genes are transiently down-regulated, and 32 genes are transiently up-regulated.

Probe sets were annotated and compiled in Gene Ontology categories using Affymetrix software [14] and the FlyBase data base [15,16] (Additional data files 3 and 4). Gene Ontology categories that are significantly more abundant among the probe sets than expected by chance include genes associated with olfactory function, which are acutely down-regulated, regulators of signal transduction, which are acutely up-regulated, metabolic enzymes, including peptidases, hydrolases and pigmentation genes, which are slowly down-regulated, and transcriptional regulators and circadian genes, which are slowly up-regulated (Additional data files 3 and 4). It is possible that the slow up-regulation of genes affecting circadian rhythm could be attributable to the two hour difference in sampling the control animals and those tested for tolerance.

Expression of a suite of genes associated with odor recognition is acutely down-regulated, including genes that encode the odorant binding proteins *lush*, *Obp19a*, *Pbprp1-5*, the odorant receptor *Or67d*, and the ubiquitous odorant receptor *Or83b*, which is necessary for transport and insertion of odorant receptors in the chemosensory dendritic membranes of olfactory neurons [17] (Table 1 and Additional data file 2). Olfactory specific protein E and antennal proteins 5 and 10 are also acutely down-regulated (Table 1). This pattern of altered transcript abundance reflects acute down-regulation of olfactory function upon exposure to undiluted ethanol vapor. Expression of two olfactory proteins is up-regulated: *Obp99d *is slowly up-regulated and *Pinocchio*, a gene that encodes a protein implicated in removal of xenobiotics from the sensillar perilymph [18], is transiently up-regulated upon exposure to ethanol (Table 1). In addition, biotransformation enzymes, including those encoded by *Cyp6a2 *and *Cyp6a13*, and *glutathione-S-transferase D5*, are acutely up-regulated. Since Pinocchio is extensively expressed throughout the third antennal segment [18], it is unlikely that the observed reduction in expression of the subset of odorant binding proteins and odorant receptors is due to ethanol induced tissue damage.

Immediate up-regulation is also observed for transcripts involved in response to stress, including *l(2)efl*, *Mpk2 *and *Hsp70Ab*. Several transcriptional regulators are rapidly up-regulated, including *cabut*, *Aly *and *Drop *along with transcripts involved in protein ubiquitination (*mib1*, *CG11414*, *CG40045 *and *TSG101*). *CG1516*, which is orthologous to human pyruvate carboxylase is also acutely up-regulated after ethanol exposure (Additional data file 2).

During the two hour period in which tolerance culminates we observe modulation of transcription of a different set of genes. Sixteen genes that encode proteases are down-regulated along with six genes associated with lipid transport and fatty acid metabolism, including *bubblegum*, *NLaz *and *Hmgs*. Two biotransformation enzymes, encoded by *Cyp12e1 *and *Cyp4ac1*, are also down-regulated, as is the transcriptional regulator *Sir2*.

At the same time there is an extensive increase in expression of transcriptional regulators, proteases and metabolic enzymes. Nineteen transcription factors, eleven proteases and thirty enzymes associated with metabolism, including signal transduction components (protein kinase C δ, *rdgA*, G protein subunit γ1, *fz2*), enzymes associated with fatty acid biosynthesis and intermediary metabolism (for example, malic enzyme), and biotransformation enzymes (encoded by *Cyp6a8*, *Cyp4e3*, *Cyp309a1*, *GstD1*, *GstE5*, *GstE7*) are up-regulated (Additional data file 2). Thus, a single exposure to ethanol has the potential to elicit profound changes in the protein composition of the cell through altered transcriptional regulation and proteolysis aimed at rapidly adapting cellular metabolism to the consequences of alcohol intoxication.

Forty-six genes with altered transcript abundance on our microarrays correspond to murine orthologues implicated in altered transcriptional regulation in a recent meta-analysis study of alcohol drinking preference in mice [19] (Additional data file 2). Furthermore, several of the genes with changes in transcript levels have been associated previously with responses to alcohol exposure, including *lush*, which encodes a *Drosophila *odorant binding protein that interacts with short chain alcohols [20] and the contact pheromone *cis*-vaccenyl acetate [21], *NLaz*, which encodes an orthologue of human apolipoprotein D, which is preferentially expressed in prefrontal cortex of alcoholics [22], and *period*, a regulator of circadian activity that has been associated with alcohol consumption in mice and humans [23]. In addition, *Sorbitol dehydrogenase 2*, *CG1600 *and *v(2)k05816*, which harbor alcohol dehydrogenase activity (Additional data file 2), also show altered transcript levels.

Mutant analysis has implicated a handful of other genes in sensitivity to ethanol. These include the *cheapdate *allele of *amnesiac *[24], which encodes a neuropeptide thought to activate the cyclic AMP signaling pathway [25]; the calcium/calmodulin-dependent adenylate cyclase encoded by the *rutabaga *(*rut*) gene [24]; the axonal migration and cell adhesion receptor, *fasciclin II *(*Fas2*) [26]; *PkaR2*, which encodes a cyclic AMP-dependent protein kinase [27]; the gene encoding the GABA-B receptor 1 [28]; and the genes encoding the *Drosophila *neuropeptide F (*npf*, a homolog of the mammalian neuropeptide Y) and its receptor [29]. In addition, heat stress has been found to induce tolerance to a subsequent exposure to ethanol and implicated a nucleic acid binding zinc finger protein encoded by the *hangover *(*hang*) gene in both the response to heat stress and the induction of ethanol tolerance [30]. Induction of ethanol tolerance was completely abolished in flies carrying both null mutations in *hang *and in the gene encoding tyramine β-hydroxylase (*Tbh*), which suggested that *hang *and the neurotransmitter octopamine mediate separate pathways involved in the induction of ethanol tolerance [30]. Further, mutations in *slowpoke*, which encodes a large-conductance calcium-activated potassium channel, eliminate the capacity for rapid tolerance, defined as reduction of the duration of sedation on a second exposure to ethanol [31,32].

However, none of these genes exhibited significant alterations in transcript abundance following one or two exposures to ethanol in this study, at our false discovery rate threshold of *q *< 0.05. Several factors could account for this discrepancy. First, the genes may have low levels of expression in adult flies, such as *amnesiac *and *GABA-B-1*, which had absent calls and were consequently not included in the analysis. Second, the magnitude of the differences in expression may have been too small to be detected with our experimental design. *Fas2*, *Pka-R2*, *rut*, *npf*, *hang *and *Tbh *all exhibited changes in transcript abundance on exposure to ethanol, but the mean differences between treatments were not large enough to be significant. Third, differences in the definition of tolerance may be relevant. *slowpoke *expression in the nervous system is required for tolerance defined as reduced sedation on a second exposure to ethanol four hours after the initial exposure [31,32] but the effects of reduced *slowpoke *expression have not been tested in our paradigm. Fourth, the mutational effects may be due to post-transcriptional regulation. Nevertheless, our results point at modulation of a complex genetic network upon alcohol exposure, in contrast to a simple linear model of two distinct and independent cellular pathways [30].

**Figure 3:**
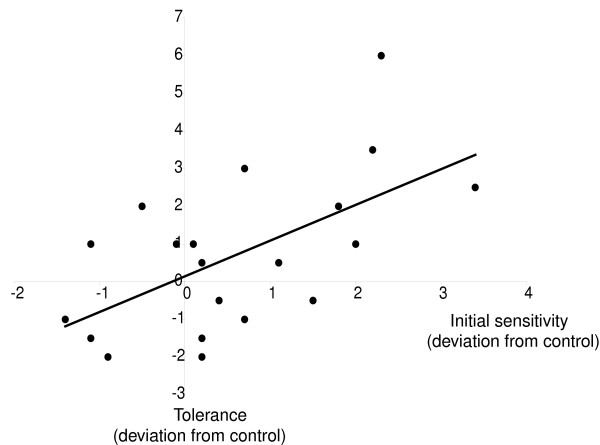
Correlation between initial sensitivity to the inebriating effects of ethanol and the subsequent development of tolerance for 20 *P*-element mutant lines. All values are expressed as deviations from the co-isogenic control line.

Next, we asked to what extent genes with altered expression on the microarrays (Additional data file 2) can be causally implicated in determining alcohol sensitivity or the tolerance response. We identified 20 genes with altered transcription following alcohol exposure for which co-isogenic *P*-element insert lines were available with a transposon insertion in or near the candidate gene [12], and determined elution profiles during the initial alcohol exposure and two hours later. Five replicate assays were performed for each line and mean elution times were compared to the appropriate co-isogenic *P*-element free control strains in either the *Canton-S *A, B, C, E or F genetic backgrounds (Table 2). One line shows greater sensitivity and seven lines are more resistant to a single alcohol exposure than the controls. Four lines show a statistically significant reduction in the peak shift of the modal elution time two hours after the initial exposure, whereas seven showed a greater shift in modal elution time after developing alcohol tolerance. It is of interest to note that three *P*-element insertion lines in the *Canton-S *E genetic background, which itself shows only a small tolerance response (Figure 1a), show substantially increased tolerance responses when transposons are inserted in or near the *Malic enzyme*, *Thor *or *fz2 *genes. Overall, there is a significant positive correlation (*r *= 0.61 ± 0.187, 0.001 <*p *< 0.01) between the mutational effects on the initial sensitivity to the inebriating effects of ethanol and the subsequent development of tolerance (Table 2, Figure 3). While mutations in genes associated with increased resistance to the first exposure to alcohol tend to develop greater tolerance (for example, *Thor*, *Malic enzyme*, *CG9248*), there are also many instances of mutations affecting only increased (for example, *Fkbp13*, *lola*) or decreased (for example, *Sir2*, *CG923*8) induction of tolerance, indicating that the development of tolerance is a process that is at least partially independent from initial sensitivity to alcohol exposure.

In total, 15 *P*-element insertion lines (75%) show altered responsiveness to ethanol exposure compared to controls (Table 2); 6 of these lines had mutational effects on both initial response and tolerance; 6 affected only tolerance, and 3 affected only the initial response. This percentage is similar to a previous study in which 67% of co-regulated genes identified on expression microarrays of single *P*-element insert *smell impaired *mutations could be implicated in an epistatic network that mediates olfactory avoidance behavior [33]. Thus, expression microarray analysis is an efficient method for identifying candidate genes affecting complex behavioral and physiological traits.

Among the 15 *P*-element [34] insertion lines that tag genes with differences in transcript abundance on exposure to ethanol, 10 have human orthologues (Table 2), including 5 enzymes associated with intermediary metabolism and fatty acid biosynthesis (*CG1516*, *Pyruvate dehydrogenase kinase*, *Malic enzyme*, *bubblegum *and *v(2)k05816 *with corresponding human orthologues *pyruvate carboxylase *(*PC*), *Pyruvate dehydrogenase kinase*, *isoenzyme 3 *(*PDK3*), *Malic enzyme 1*, *NADP(+)-dependent *(*ME1*), *bubblegum related protein *(*BGR*), and *fatty acid synthase *(*FASN*)), three transcription factors (*elbow B*, *spalt major *and *frizzled 2 *with corresponding human orthologues *zinc finger protein 503 *(*ZNF503*), *sal-like1 *(*SALL1*) and *frizzled homolog 8 *(*FZD8*)), *CG9086 *(with human orthologue *Ubiquitin protein ligase E3 component n-recognin 2 *(*UBR2*)), and *nuclear fallout *(with human orthologue *RAB11 family interacting protein 4 *(*RAB11FIP4*)).

**Figure 4:**
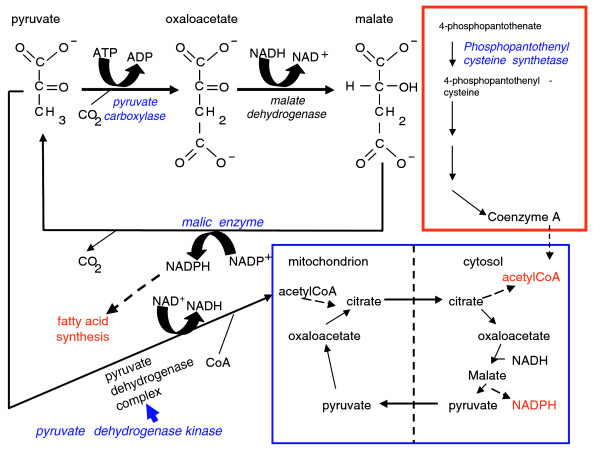
Identification of a conserved metabolic network likely to be associated with alcohol sensitivity in flies and humans. Ethanol is converted via the alcohol dehydrogenase and aldehyde dehydrogenase reactions into acetate, which is subsequently conjugated to co-enzyme A (CoA; not shown). The biosynthetic pathway of co-enzyme A is schematically depicted in the red box. Acetyl-CoA produced in excess can be converted into fatty acids. The diagram highlights auxiliary pathways for the biosynthesis of fatty acids. The blue box illustrates how pyruvate carboxylase and malic enzyme mediate a cyclic metabolic pathway, which via the mitochondrial citrate and pyruvate transporters results in the net transport of acetyl-CoA across the mitochondrial membrane and generation of cytosolic NADPH, both critical substrates for fatty acid metabolism. An alternative metabolic pathway is the direct conversion of pyruvate into acetyl-CoA via the pyruvate dehydrogenase complex. This complex is inhibited through phosphorylation by pyruvate dehydrogenase kinase.

Alcohol-induced fatty acid biosynthesis is well documented in heavy drinkers and leads to alcohol induced fatty liver syndrome in alcohol-dependent people [35,36]. The identification of multiple enzymes associated with intermediary metabolism and fatty acid biosynthesis in the response to alcohol exposure in *Drosophila *is, therefore, of particular interest. Pyruvate carboxylase, malic enzyme and pyruvate dehydrogenase kinase are critical regulators of intermediary metabolism (Figure 4). Another gene with altered transcriptional regulation, *CG5629*, is also of interest, even though no homozygous viable co-isogenic *P*-element insertion line is available. *CG5629 *encodes an orthologue of phosphopantothenyl cysteine synthetase, which is critical for fatty acid biosynthesis as it participates in the biosynthesis of Coenzyme A. Genes associated with pyruvate metabolism, including pyruvate carboxylase and pyruvate dehydrogenase kinase, also showed altered transcriptional regulation in alcohol preferring inbred mice [19]. These observations point to a central role for pyruvate metabolism in determining alcohol sensitivity and suggest that the cyclic metabolic pathway, which enables transport of acetyl-CoA across the mitochondrial membrane and generation of cytosolic NADPH, both critical substrates for fatty acid metabolism, is an important determinant for the ability to develop alcohol tolerance (Figure 4).

## Conclusion

*Drosophila *provides a powerful model for the identification of genes that respond to alcohol exposure. Dependent on their genetic background, flies can develop tolerance after an initial exposure, and transcriptional profiling shows that the development of tolerance is a process that is at least partially independent from initial sensitivity to alcohol. A significant fraction of genes implicated in the response to alcohol in flies have human orthologues and form part of conserved cellular and metabolic pathways. Whereas most studies on alcoholism in human populations have targeted genes associated with neurotransmission, our findings from the *Drosophila *model suggest that it might be fruitful to refocus some of these efforts on other targets, including (but not limited to) biotransformation pathways, transcriptional regulators, proteolysis and enzymes that act as metabolic switches in the regulation of fatty acid metabolism.

## Materials and methods

### *Drosophila *stocks

Flies used for microarray experiments were in an isogenic *Canton-S *B genetic background. Homozygous *P*-element insertion lines contained *P(GT1)-*elements [34] in or near candidate genes in co-isogenic *Canton S *A, B, C, E or F backgrounds and were generated by Dr Hugo Bellen (Baylor College of Medicine, Houston, TX, USA) as part of the Berkeley *Drosophila *Genome Project [12]. Behavioral assays were conducted between 9:00 am and 1:00 pm. Flies were not exposed to CO_2 _anesthesia for at least 24 h prior to the assay. All flies were reared on cornmeal/molasses/agar medium under standard culture conditions (25°C, 12:12 hour light/dark cycle).

### Alcohol sensitivity and tolerance

To quantify alcohol sensitivity, we tested flies (5 replicates/genotype, *N *= 100/replicate) in an inebriometer [11] pre-equilibrated with ethanol vapor, from which they were eluted at one minute intervals. The mean elution time is a measure of alcohol sensitivity. To measure alcohol tolerance [10], we subjected flies to an initial exposure to ethanol, and recorded their elution profile as described above. We allowed the flies to recover for 2 h, and then repeated the assay with the same flies. The difference in the shift of modal elution times (5 replicates/genotype, *N *= 100/replicate) between the second and first assays is a measure of tolerance. We used analysis of variance to assess whether the sensitivity of *P*-element insertion lines differed significantly from the control, according to the model:

Y = *μ *+ *L *+ *R*(*L*) + *ε*

where *μ *is the overall mean, *L *is the fixed effect of line (*P*-element insertion versus control), *R *is the random effect of replicate, nested within line, and *ε *is the variance within replicates. We used *t*-tests to assess the significance of the difference in modal elution times between the first and second exposures.

### Whole genome expression analysis

To assess transcriptional regulation after ethanol exposure, we used 3 to 5 day old *Canton-S *B males. We collected five replicates of pools of 30 flies with 0, 1 or 2 exposures to ethanol. For each replicate, we began with two groups of >100 flies. A total of 15 flies from each group was not exposed to ethanol (control flies; Figure 1b) and were pooled and frozen immediately on dry ice. We passed the remaining flies from each group through the inebriometer, and collected individuals that eluted at 3 to 5 minutes (the peak elution time for the first exposure; Figure 1b). A total of 15 of these flies from each group was pooled and frozen immediately on dry ice. The remaining flies were kept on standard fly food for 2 h. They were then passed once again through the inebriometer in 2 groups, and 15 flies from each group that eluted at 7 to 10 minutes (the peak elution time for the 2nd exposure; Figure 1b) were collected, pooled, and frozen.

Total RNA was extracted from the 15 samples (three treatments × five replicates/treatment) using the Trizol reagent (Gibco BRL, Gaithersburg MD, USA). Biotinylated cRNA probes were hybridized to high density oligonucleotide microarrays (*Drosophila *GeneChip 2.0, Affymetrix Inc., Santa Clara, CA, USA) and visualized with a streptavidin-phycoerythrin conjugate, as described in the Affymetrix GeneChip Expression Analysis Technical Manual (2000), using internal references for quantification.

### Data analysis

The quantitative estimate of expression of each probe set is the *Signal *(*Sig*) metric, as described in the Affymetrix Microarray Suite, Version 5.0. The 18,769 probe sets on the Affymetrix *Drosophila *GeneChip 2.0 are represented by 14 perfect-match (PM) and 14 mismatch (MM) pairs. The *Sig *metric is computed using the weighted log(PM-MM) intensity for each probe set, and was scaled to a median intensity of 500. A detection call of Present, Absent, or Marginal is also reported for each probe set. We excluded all probe sets with samples called 'Absent' from the analysis. On the remaining probe sets, we conducted one-way fixed effect ANOVAs of the *Signal *metric using the GLM procedure in SAS [37], with the following model:

*Y *= *μ *+ *T *+ *ε*

where *Y *is the observed value for each treatment, *μ *is the overall mean value, *T *is the fixed effect of treatment and *ε *is the error variance between replicate arrays. We corrected the *p *values for multiple tests by calculating the false positive discovery rate *q*-value, which estimates the proportion of false positives among all terms declared significant [13] and used a confidence level of *q *< 0.05 as measure of statistical significance. We identified 582 probe sets with altered transcriptional regulation at *q *< 0.05 (Additional data file 2) and performed further analysis by *post hoc *Tukey tests using the MEANS procedure in SAS [37] to identify the direction (up- or down-regulation) of expression between treatment groups (control, 1st exposure, and 2nd exposure) at the *p *< 0.05 level. Gene ontology categories were annotated using Affymetrix [14] and FlyBase [15,16] compilations. We conducted χ^2 ^tests to determine which categories are over- or under-represented by probe sets that are significantly up- or down-regulated between treatment groups compared to the expected number of genes in each category based on its representation on the microarray (Additional data files 3 and 4). The microarray data discussed in this publication have been deposited in NCBIs Gene Expression Omnibus [38] and are accessible through GEO Series number GSE5382 [39].

## Additional data files

The following additional data are available with the online version of this paper. Additional data file 1 contains raw microarray data without any analysis. Additional data file 2 is a list of all probe sets differentially expressed between treatment groups with *q *< 0.05. Additional data file 3 contains biological process gene ontology categories of the genes listed in Additional data file 2. Additional data file 4 contains molecular function gene ontology categories of genes listed in Additional data file 2. Additional data file 5 lists information regarding influences of different genetic backgrounds on ethanol sensitivity and tolerance development.

## Supplementary Material

Additional data file 1 Raw microarray data without any analysis. Click here for file

Additional data file 2 List of all probe sets differentially expressed between treatment groups with *q *< 0.05. Click here for file

Additional data file 3 Biological process gene ontology categories of the genes listed in Additional data file 2. Click here for file

Additional data file 4 Molecular function gene ontology categories of genes listed in Additional data file 2. Click here for file

Additional data file 5 Influences of different genetic backgrounds on ethanol sensitivity and tolerance development. Click here for file

## Figures and Tables

**Table 1:** Probe sets with altered transcriptional regulation after ethanol exposure with *q *< 0.001

Probe set ID	*p *value	Gene name	Biological process	Changes in expression
1623332_at	1.91E-05	*CG31106*	Transmission of nerve impulse; carbohydrate metabolism	Transient up-regulation
1623605_a_at	2.20E-05	*Serpin-27A *(M,H)*	Toll signaling pathway; defense response	Transient up-regulation
1624662_at	2.20E-05	*CG11414*	Protein ubiquitination	Acute up-regulation
1624736_a_at	2.20E-05	*CG11878*	Unknown	Slow up-regulation
1625193_at	2.20E-05	*CG31272*	Lipid metabolism; neurotransmitter secretion; transport	Slow up-regulation
1626882_at	2.35E-05	*Pinocchio*	Olfactory behavior;response to chemical stimulus	Transient up-regulation
1627080_at	5.07E-05	*CG14893*	Unknown	Slow up-regulation
1627385_at	6.86E-05	*minidiscs*	Amino acid transport, metabolism	Transient down-regulation
1628229_at	6.86E-05	*Trypsin 29F*	Proteolysis	Slow up-regulation
1628494_a_at	6.86E-05	*CG33525*	Negative regulation of transcription, DNA-dependent	Slow up-regulation
1628647_at	1.06E-04	*CG33110*	Fatty acid biosynthesis; very-long-chain fatty acid metabolism	Slow up-regulation
1628947_s_at	1.06E-04	*Ecdysone-inducible gene L2 *(M,H)*	Cell adhesion	Acute up-regulation
1629012_at	1.06E-04	*period *(M,H)*	Long-term memory; circadian rhythm; signal transduction; negative regulation of transcription from RNA polymerase II promoter	Transient down-regulation
1629592_at	1.32E-04	*ηTrypsin *(M,H)*	Proteolysis	Transient down-regulation
1629617_at	1.44E-04	*cabut*	Regulation of transcription from RNA polymerase II promoter; JNK cascade; autophagic cell death	Acute up-regulation
1630302_at	1.66E-04	*Odorant-binding protein 99d*	Transport; autophagic cell death	Slow up-regulation
1631406_at	1.66E-04	*CG3106 *(M,H)*	Unknown	Transient down-regulation
1632222_a_at	2.25E-04	*CG17752*	Extracellular transport; cation transport	Slow up-regulation
1632228_at	2.58E-04	*CG10505*	Response to toxin; extracellular transport; defense response	Slow up-regulation
1633700_at	2.78E-04	*antennal protein 10 *(M,H)*	Sensory perception of chemical stimulus	Acute down-regulation
1633794_a_at	3.22E-04	*antennal protein 5*	Signal transduction	Acute down-regulation
1633913_at	3.80E-04	*Cytochrome P450-6a8*	Electron transport; steroid metabolism; lauric acid metabolism	Slow up-regulation
1634318_at	3.98E-04	*lush*	Response to ethanol; sensory perception of smell; olfactory behavior	Acute down-regulation
1634591_at	4.38E-04	*Rpd3*	Regulation of transcription from RNA polymerase II promoter; determination of adult life span; gene silencing	Slow up-regulation
1635815_at	5.47E-04	*CG5819 *(M,H)*	Transmission of nerve impulse; cell adhesion	Acute up-regulation
1636202_s_at	6.40E-04	*CG8931*	Transport	Transient up-regulation
1636611_at	6.40E-04	*Pheromone-binding protein-related protein 1 *(M)*	Signal transduction; sensory perception; response to pheromone	Acute down-regulation
1636771_at	6.40E-04	*CG8562*	Proteolysis	Slow down-regulation
1638066_at	6.40E-04	*CG9238*	Glycogen metabolism	Slow up-regulation
1638246_at	6.52E-04	*Olfactory-specific E*	Signal transduction; response to pheromone; transport	Acute down-regulation
1638452_at	7.01E-04	*CG6733*	Proteolysis	Slow down-regulation
1639368_at	7.73E-04	*CG5804*	Lipid transport; cell acyl-CoA homeostasis	Transient down-regulation
1639934_at	7.77E-04	*capulet*	Actin filament organization; bristle morphogenesis	Slow up-regulation
1640755_at	8.83E-04	*Picot*	Phosphate metabolism; extracellular transport; cation transport	Slow up-regulation

Transcripts that show sustained altered expression after the first exposure to ethanol are indicated as 'acutely up- or down-regulated genes'. Transcripts that show altered expression only after the second exposure are designated as 'slowly up- or down-regulated genes'. The designation 'transient up-regulation' refers to genes that show increased expression after the first exposure to ethanol, but a return to control levels 2 h later after the second exposure. *Genes that have human (H) and/or mouse (M) orthologues.

**Table 2:** *P[GT1]*-element insertion lines with transposon insertions at genes with ethanol-dependent altered transcriptional regulation

Gene symbol	*P*-element insertion site	Biological process	Canton S* background	Mean elution time (min)^† ^(1st exposure)	Time shift (between 1st and 2nd exposures)
**Slow down-regulation**					
*Fkbp13*	In 1st intron	Protein folding	A	6.7 ± 0.25	2 min^‡ ^↑
*Sir2*	410 bp in exon 1	Regulation of transcription, DNA-dependent; chromatin silencing	B	5.0 ± 0.2	2 min^§ ^↓
*Bubblegum*^*H*^	In 1st intron	Fatty acid metabolism	C	6.2 ± 0.21	0.5 min (NS) ↓
**Acute up-regulation**					
*CG1516*^*H*^	125 bp upstream of transcription initiation site of the longest transcript	Pyruvate metabolism; fatty acid biosynthesis	E	4.2 ± 0.12	1 min (NS) ↑
**Transient up-regulation**					
*Thor*	431 bp upstream of transcription initiation site	Negative regulation of translational initiation; response to stress; defense response	E	7.5 ± 0.11; R	2.5 min^§ ^↑
*Pyruvate dehydrogenase kinase*^*H*^	47 bp in exon 1	Pyruvate metabolism; tricarboxylic acid cycle; regulation of phosphate metabolism	F	7.4 ± 0.30; R	1 min^‡ ^↑
**Slow up-regulation**					
*v(2)k05816*^*H*^	281 bp upstream of transcription initiation site	Fatty acid biosynthesis	A	6.1 ± 0.25	1 min (NS) ↑
*elbow B*^*H*^	888 bp upstream of transcription initiation site	Tracheal system development	A	6.8 ± 0.26	0.5 min (NS) ↓
*Hormone receptor-like in 38*	1,428 kb upstream of transcription initiation site	Regulation of transcription from RNA polymerase II promoter; intracellular signaling cascade	A	5.8 ± 0.22; S	1 min (NS) ↓
*nuclear fallout*^*H*^	In 4th intron	Actin cytoskeleton reorganization; microtubule-based process	B	6.6 ± 0.30	1 min (NS) ↓
*CG12505*	136 bp upstream of transcription initiation site	Unknown	B	4.8 ± 0.18	1.5 min^‡ ^↓
*tramtrack*	219 bp in exon 1 of the longest transcript	Regulation of transcription from RNA polymerase II promoter; peripheral nervous system development	B	5.8 ± 0.20	1 min^§ ^↑
*CG9086*^*H*^	50 bp in exon 1	Protein ubiquitination	B	6.1 ± 0.18	1.5 min^§ ^↓
*CG6767*	In 1st intron	Nucleotide biosynthesis, metabolism	B	7.0 ± 0.16; R	0.5 min (NS) ↑
*CG32434*	36 bp upstream of transcription initiation site	Unknown	B	6.1 ± 0.26	2 min^§ ^↓
*frizzled 2*^*H*^	147 bp in exon 1	Signal transduction; G-protein coupled receptor protein signaling pathway	E	5.9 ± 0.13; R	2 min^§ ^↑
*Malic enzyme*^*H*^	In 1st intron	Malate metabolism; tricarboxylic acid cycle	E	6.4 ± 0.15; R	6 min^¶ ^↑
*CG9238*	73 bp in exon 1	Glycogen metabolism	F	7.6 ± 0.12; R	3.5 min^§ ^↑
*spalt major*^*H*^	154 bp upstream of transcription initiation site	Tracheal system development; sensory organ development; negative regulation of transcription	F	6.9 ± 0.18; R	0.5 min (NS) ↓
*lola*	In 1st intron	Transmission of nerve impulse; regulation of transcription from RNA polymerase II promoter	F	6.1 ± 0.11; R	3 min^§ ^↑

*See Additional data file 5 for mean elution times of *P*-element free Canton S genetic backgrounds A, B, C, E and F. ^†^S and R designate *P*-element insert lines that are significantly more sensitive or more resistant than the co-isogenic control, respectively (one-way ANOVA). An *H *after a gene symbol indicates genes that have human orthologues. Up and down arrows designate increase or decrease, respectively, in the time shift between the first and second ethanol exposure compared with the co-isogenic control (Student's *t*-test). ^‡^*p *< 0.05, ^§^*p *< 0.01, ^¶^*p *< 0.001. bp, base-pairs; NS, not significant.
