# Supplementary material for: Transcriptional response to alcohol exposure in Drosophila melanogaster
Source: Genome Biol. 2006 Oct 20;7(10):R95. doi: 10.1186/gb-2006-7-10-r95 (PMC1794562; doi:10.1186/gb-2006-7-10-r95)
Supplement: Additional data file 5 — Influences of different genetic backgrounds on ethanol sensitivity and tolerance development. [file gb-2006-7-10-r95-S5.doc]

| **Supplementary Table 1. Influences of different genetic background on ethanol sensitivity and tolerance development.** | | |
| --- | --- | --- |
|  |  |  |
| ***Canton S* Background** | **Mean elution time (min) (1^st^ exposure)** | **Time shift (min) (between 1^st^ and 2^nd^ exposures)** |
| **A** | 7.2 ± 0.12 | 2 min, * |
| **B** | 5.9 ± 0.04 | 4 min, *** |
| **C** | 6.0 ± 0.15 | 2 min, ** |
| **E** | 4.1 ± 0.07 | 1 min, ns |
| **F** | 5.4 ± 0.07 | 1 min, ns |
| **3-5 day old flies, *Canton S* B** | 5.5 ± 0.05 | 4 min, *** |
| **12-13 day old flies, *Canton S* B** | 7.5 ± 0.11 | 2 min, ** |
| * P < 0.05, ** P < 0.01, *** P < 0.001; ns, not significant. | | |
